# Population Dynamics of Bulking and Foaming Bacteria in a Full-scale Wastewater Treatment Plant over Five Years

**DOI:** 10.1038/srep24180

**Published:** 2016-04-11

**Authors:** Xiao-Tao Jiang, Feng Guo, Tong Zhang

**Affiliations:** 1 Environmental Biotechnology Lab, The University of Hong Kong SAR China.

## Abstract

Bulking and foaming are two notorious problems in activated sludge wastewater treatment plants (WWTPs), which are mainly associated with the excessive growth of bulking and foaming bacteria (BFB). However, studies on affecting factors of BFB in full-scale WWTPs are still limited. In this study, data sets of high-throughput sequencing (HTS) of 16S V3–V4 amplicons of 58 monthly activated sludge samples from a municipal WWTP was re-analyzed to investigate the BFB dynamics and further to study the determinative factors. The population of BFB occupied 0.6~36% (averagely 8.5% ± 7.3%) of the total bacteria and showed seasonal variations with higher abundance in winter-spring than summer-autumn. Pair-wise correlation analysis and canonical correlation analysis (CCA) showed that *Gordonia* sp. was positively correlated with NO_2_-N and negatively correlated with NO_3_-N, and Nostocodia limicola II *Tetraspharea* sp. was negatively correlated with temperature and positively correlated with NH_3_-N in activated sludge. Bacteria species correlated with BFB could be clustered into two negatively related modules. Moreover, with intensive time series sampling, the dominant BFB could be accurately modeled with environmental interaction network, i.e. environmental parameters and biotic interactions between BFB and related bacteria, indicating that abiotic and biotic factors were both crucial to the dynamics of BFB.

Bulking and foaming are two operational problems in activated sludge (AS) wastewater treatment plants (WWTPs)^1^,^2^. Bulking affects the settleability of bioflocs, which may result in failure of solid-liquid separation^3^ while foaming on the water surface of aeration tank needs extra operation, lowers the effluent quality and causes loss of biomass^4^. Even though these issues have experienced extensive amount of research by both improving configuration of process and controlling the relevant filamentous bacteria, there is still no systematic method to treat them and they still occur sporadically all over the world^5^,^6^,^7^. The bulking and foaming bacteria (BFB) are deemed as those bacteria overgrowth in a sludge bulking or foaming episode. Their roles in sludge bulking or foaming are not well studied, although their physical roles in the floc formation are well documented as the backbone of flocs in AS^7^,^8^,^9^.

Morphological standards were established to identify BFB by microscopy and chemical staining^2^, and then various molecular based methods such as T-RFLP^10^, DGGE^11^, real-time PCR^12^,^13^ and FISH^7^ were applied to survey the existence and abundance of BFB temporally or spatially. However, very limited associations between physiochemical, operational parameters and BFB were detected which possibly due to the limited identification accuracy of morphological methods, low throughput of T-RFLP, DGGE or poor quantification of FISH^5^. In recent years, large-scale microbial communities profiling with high throughput sequencing (HTS) of 16S rRNA amplicons^14^,^15^ and whole environmental DNA^16^,^17^ have become powerful tools to investigate microbes in environmental samples. Pair-wise correlation (Mostly Pearson or Spearman correlation) based network analyses among microbial communities and environmental parameters by HTS of 16S rRNA markers in various environmental niches such as soil, human gut and AS have been conducted to reveal environment-microbe and microbe-microbe associations, which have deepen our understanding of the determinative factors and the taxonomic relatedness on microbial communities^18^,^19^,^20^. This method could explain the possible linear (Person) or rank linear (Spearman) relationships between microbial communities and environmental parameters. For nonlinear relationships, the environmental interaction network (EIN) method had been proven as an effect way to model the dynamics of microbial assemblages in Western English Channel ocean region^21^, which is different from other modeling methods in WWTPs which focused on prediction of effluent quality and sludge volume index (SVI) with physicochemical parameters and operational parameters.

To the best of our knowledge, currently there are no studies using large-scale HTS of 16S rRNA marker time series data to specifically investigate the associations between abiotic (environmental parameters), biotic (other related bacteria) factors and BFB. In this study, monthly activated sludge samples had been collected from Shatin WWTP in Hong Kong S.A.R. over five years (2007~2012) and the temporal dynamics of the overall bacterial communities has been investigated in our previous study^22^. The aims of this study were to 1) profile all identified BFB dynamics in a full-scale WWTP over five years using HTS of 16S rRNA amplicons, 2) explore the correlations between BFB and environmental parameters as well as other bacterial taxa, and 3) model BFB dynamics with environmental parameters and interactions between BFB and related bacteria using EIN.

## Results and Discussion

### Identification and quantification of bulking and foaming bacteria

**Figure 1:**
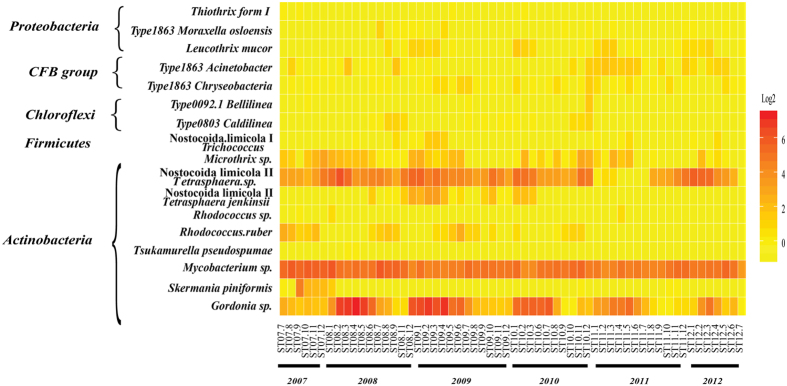
Heatmap of bulking and foaming bacteria over 58 months from 2007 to 2012 at similarity of 97% (sequences with hit length less than 300 bps were removed and not counted). Heatmap values were transformed to log2. Bulking and foaming bacteria were grouped with phyla.

In this study, 16S V3–V4 pyrosequencing data sets^22^ of 58 samples collected over five years from 2007 to 2012 were re-analyzed for BFB profiles. In Hong Kong people use sea water to do toilet flushing, as a result, the municipal wastewater treated in the Shatin WWTP contains about 30% sea water and is high in salinity. Over the whole sampling period, the WWTP was efficient in CBOD removal however suffered by unstable ammonium removal and periodically foaming in winter each year^22^. After normalizing the sequencing depth to 6000 sequences for each sample, 384 K sequences were obtained totally. The sequences were then aligned to BFB database with BLAST 2.28+ to identify BFB by a similarity of 97% and a hit length over 300 bps^15^. The BFB database contained the full-length 16S rRNA sequences of bacteria which were reported in literatures as the bacteria responsible for AS bulking or foaming^15^. The abundances of each BFB type were the sum of 16S rRNA sequences abundance with 97% similarity aligned with the sequences in BFB database. As shown in Fig. 1, totally there were 17 types of BFB belonging to five phyla: *Actinobacteria*, *CFB* sp., *Choloroflexi*, *Firmicutes* and *Proteobacteria*, respectively. The most abundant foaming bacteria was *Gordonia* sp. (averagely 3.6% ± 6.65% of total bacteria) followed by *Mycobacterium* sp. (3% ± 1.62%). The most abundant bulking bacteria was Nostocoida.limicola II *Tetrasphaera* sp. (1.6% ± 1.8%) followed by *Microthrix* sp. (0.07% ± 0.09%). The four most abundant BFB were all from phylum *Actinobacteria*. Apart from the dominant four types, other BFB were presented sporadically across all the time with relatively low abundance.

The occurrence and abundance of BFB in Shatin WWTP were different from those documented in other studies about the diversity and abundance of BFB in different WWTPs at different countries^1^,^23^,^24^. In a survey of Italy WWTPs, *Microthrix pavicella* was the dominant filamentous microorganism involved in sludge bulking or foaming^23^; however, in the present study, it was *Gordonia* sp. which was proved to be a novel species of genus *Gordonia*^17^. For Shatin WWTP, foaming only happened in winter while sludge bulking happens at different seasons as indicated by sludge volume index (SVI). The frequency distribution graph (Fig. S1) of the dominant six BFB showed quite different frequency patterns. The most abundant species *Gordonia* sp. was present in all months. However, the abundance distribution was quite uneven with >60% months less than 1% of total bacteria and >10% months with 12~37% of total bacteria. *Mycobacterium* sp. and *Tetrasphaera* sp. had relatively stable abundance around 3.0% and 1.6% of total bacteria, respectively. The abundance pattern was different from the results of a previous survey of Danish WWTPs using quantitative FISH which showed that only minor changes in relative abundance over three years^7^. These Danish WWTPs were only suffered by minor operational problems of sludge bulking and foaming and the temperature in Danish was 7–20 °C, but the climate in Hong Kong was much warmer and temperature was 13–30 °C, which possibly was related with bigger variance of BFB (Fig. S5). The abundance of BFB in Shatin over five years had bigger variations compared with that in our previous studies of 14 sewages plants^15^ which were from 1.86 to 8.99%. Noticeably, the Shatin WWTP was included in the 14 sewages plants. The discrepancy indicated that the abundance variations of the bacteria related with bulking and foaming in Shatin WWTP over time which suffered from periodical sludge bulking and foaming in winter and spring could be larger than that from geographically distributed WWTPs from different countries.

The complexity of different frequency patterns for different BFB revealed the generalist and specificity of BFB along time. *Gordonia* sp. and *Mycobactrium* sp. were quite general since they presented in all time points, however *Gordonia* sp. simultaneously showed quite strong specificity due to the large variation of abundance along time. The advantage of this method compared with traditional method like FISH was that we can study all the potential BFB in one run as long as they were in the BFB database. Noticeably, for the identification of BFB, although we strictly controlled the criteria with a hit length of at least 300bp and the identity to 97%, the identification accuracy maybe influenced by the limited length of 16S V3-V4 region and those BFB not incorporated into the database could not be detected^15^,^25^. However, these limitations could be alleviated with the increase of sequencing length or application of third generation sequencing platform which generates sequences longer than full length of 16S rRNA^26^. At the same time, the BFB database could be more completed as more BFB were discovered. Thus, the identification of BFB using 16S profiles will be further applied in the future studies.

### Seasonal variations of bulking and foaming bacteria

**Figure 2:**
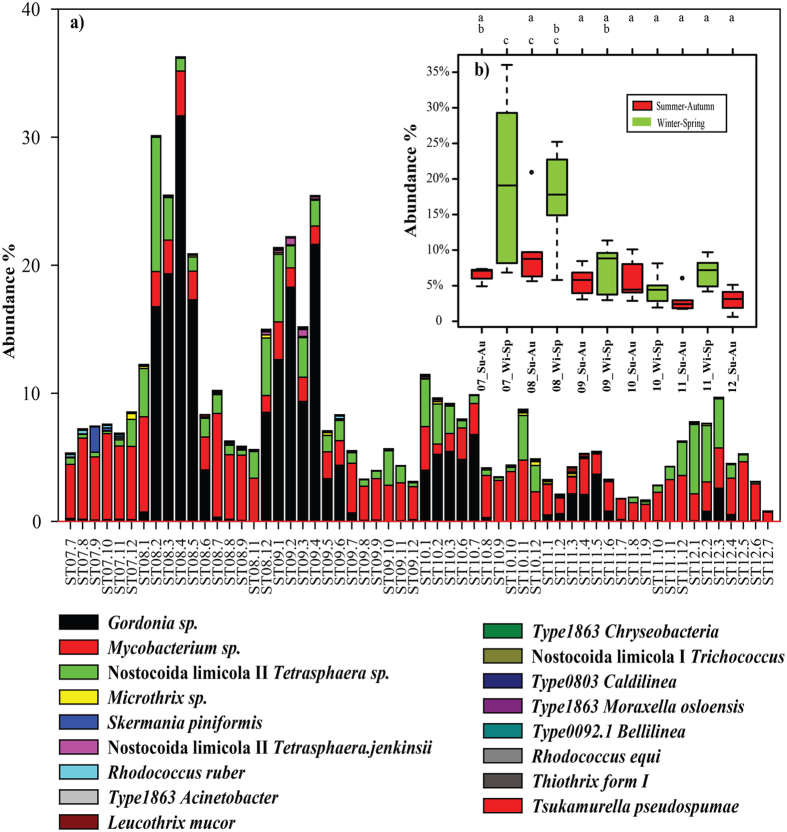
(**a**) Bar plot of the abundance of all the 17 hit BFB from July 2007 to July 2012 in activated sludge of Shatin WWTP. (**b**) Seasonal variation of total BFB over five years, winter-spring (November–April) vs. summer-autumn (May–October) and a, b, c labeled the post-hoc Tukey HSD test state among seasons, with the same label means do not have significant difference (*P*-value, 0.05), different labels represent significant difference.

Overall, total BFB showed seasonal variations with higher abundance in winter-spring and relatively lower abundance in adjacent summer-autumn except for 2010 over the five years, which was mainly contributed by the variation of *Gordonia* sp. (Fig. 2a). One way ANOVA of the summer-autumn month (May to Oct.) and winter-spring months (Nov. to Feb.) showed significant difference of total BFB over the sampling time (F-value 6.21, P-value < 0.001, Fig. 2b). Post-hoc Tukey HSD test was performed for each pair of seasons. Specifically, winter-spring months of 2007 and 2008 were significantly (P-value < 0.05) different from other time points, as indicated by different labels ‘a’, ‘b’ or ‘c’ in Fig. 2b. For each BFB, in details, *Gordonia* sp. showed huge variations, i.e. high in winter-spring months (around March and April) and low in summer months (around July and August), while *Mycobacterium* sp. showed much less variations (Fig. 1, Fig. S1 and Fig. 2a). An annual decline trend of the portion of total BFB in winter was also observed (Fig. 2b). There were sludge foaming happened in spring time around March at Shatin WWTP^22^. Although bacteria *Gordonia amarae* has been reported largely associated with sludge foaming^27^, in the present study, the existence of *Gordonia* sp. all the time before and after foaming accidents may indicate that existence of *Gordonia* sp. was not the deterministic or sole reason for the foaming accident. The temporal dynamics of total BFB was very obvious compared with a three years’ survey in 28 Danish WWTPs, which showed that only minor changes were observed for most of BFB^7^. The discrepancies between these two studies could be due to influent characteristics, climate conditions of the two regions, and operational parameters.

### Correlation between BFB and operational parameters

**Figure 3:**
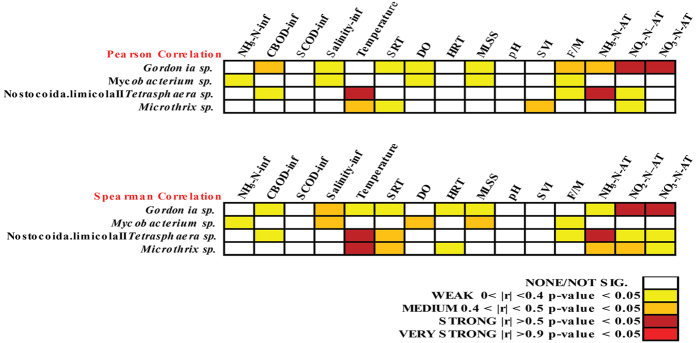
Pearson and Spearman coefficient index of the most dominant four BFB with operational parameters. Parameters with “inf” suffix were parameters of influent wastewater and parameters with “AT” suffix were aeration tank activated sludge parameters.

To investigate the influence of operational parameters on the dynamics of BFB, Pearson and Spearman coefficient indexes were calculated between the most abundant four BFB and operational parameters. In the present study, several strong correlations have been identified by correlation analysis, including *Gordonia* sp. with NO_2_-N-AT (aeration tank) and NO_3_-N-AT, *Tetraspheara* sp. with temperature and NH_3_-N-AT (Fig. 3). These correlations have never been reported before. The above results were confirmed by Canonical corresponding analysis (CCA) results, which showed that *Gordonia* sp. was positively correlated with NO_2_-N-AT (aeration tank) and negatively correlated with NO_3_-N-AT; *Tetrasphaera* sp. was negatively correlated with temperature and positively correlated with NH_3_-N-AT (Fig. S2). There were reports showed new strains in genus *Gordonia* could be nitrate reducing bacteria^28^,^29^, and this indicated that the *Gordonia* sp. was possibly related with nitrogen metabolism. We analyzed the draft genome of the novel *Gordonia* sp. and found that it was a nitrate reduction bacterium and could perform nitrate reduction by transforming nitrate into ammonia (Fig. S4). The negative correlation between *Tetrasphaera* sp. and temperature has not been reported elsewhere so it was firstly shown in this study, further genome information and metabolic information were needed to investigated the underneath reasons for the correlation. The dependence of foaming bacteria *Microthrix parvicella* on temperature had been observed in previous study^30^ which was consistent with our result. It was notable that the SVI values, as the indicator of the sludge settling ability, was only medium correlated (|Pearson correlation coefficient| <0.5) with *Microthrix* sp., implying the complex of sludge bulking (Figs 3 and S2). Due to the limitation of technologies used before, it was difficult to conduct large-scale correlation analysis of filamentous bacteria with operational parameters to identify the sensitive parameters. In the present study, we obtained several strong correlations of the operational parameters with specific BFB by the statistical analysis based on long-term time series profiling of the bacterial community in the full-scale WWTP, although these novel relationships need to be further confirmed by experiments. We also preliminarily demonstrated that the correlation of *Gordonia* sp. with nitrate, nitrite and ammonia was possibly due to that this bacterium was related with nitrogen metabolism based on the draft genome gene contents.

### Bacterial species correlated with bulking and foaming species

**Figure 4:**
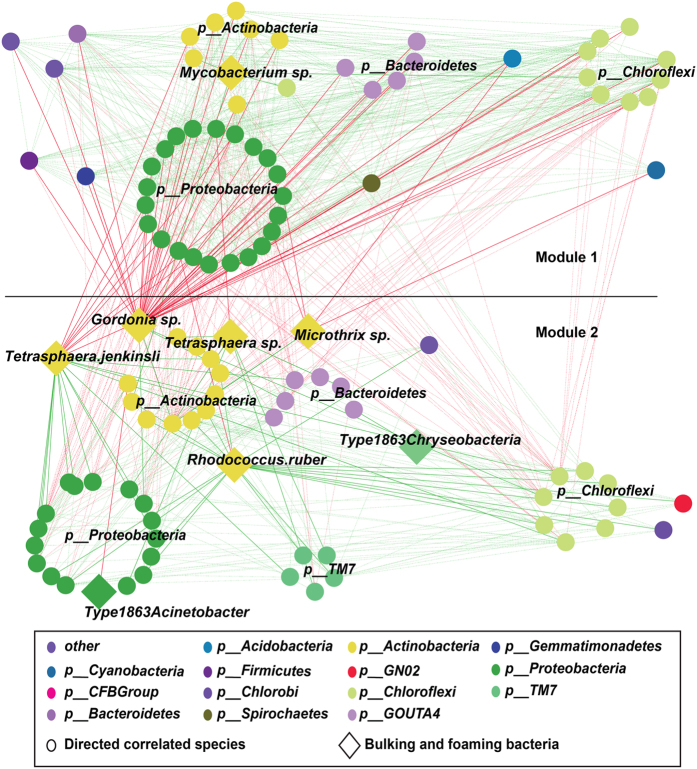
Correlation based network analysis of those bacterial species significantly correlated with BFB (spearman correlation coefficient index over 0.6 or smaller than –0.6). Nodes were bacteria and edges were the correlation between bacteria. Green edges represented positive correlation and red edges were negative correlation. All the correlated bacteria were clustered into two co-exclusive modules.

Apart from the abiotic factors, biotic interactions were also quite important for the dynamics of bacteria assemblages^21^,^22^. Bacterial species strongly correlated (|Spearman coefficient| >0.6, *P*-value < 0.01) with BFB were clustered into two modules (Fig. 4). Inside each module, bacteria species were all positively correlated; between the two modules, they are negatively correlated. The two modules both were composed with widely distributed phyla. Detailed analysis of the two modules showed that 90.3% of the species strongly correlated with *Gordonia* sp. were in Module 1 and these species were mainly from phyla *Proteobacteria* and *Chloroflexi.* Specifically, seven species from order *Rhizobiales* were all strongly and negatively correlated with *Gordonia* sp. (Fig. 4, Table S1). The species directly correlated with *Tetrashpaera* sp. and *Rhodococcus rubers* were mostly in Module 2 with positive correlation. Notably, species strongly correlated with BFB from the phylum *TM_7* were all in Module 2, which may indicate that this phylum had similar variation as BFB in Module 2.

The non-randomly modality of bacteria correlated with BFB and almost all BFB (except *Mycobacterium* sp.) belong to Module 1 indicated that different BFB populations may exhibit similar preference for environment. Generally, in microbe-microbe interaction, negative correlation may be caused by prey-predator, competition, amensalism, different preference in living environment and so on^31^. Although it is not an easy task to find the reasons for these correlation patterns in the network, the non-random positive and negative correlation of different bacterial species with BFB pose novel knowledge to BFB related associations in AS system.

### Modeling BFB dynamics with EIN

**Figure 5:**
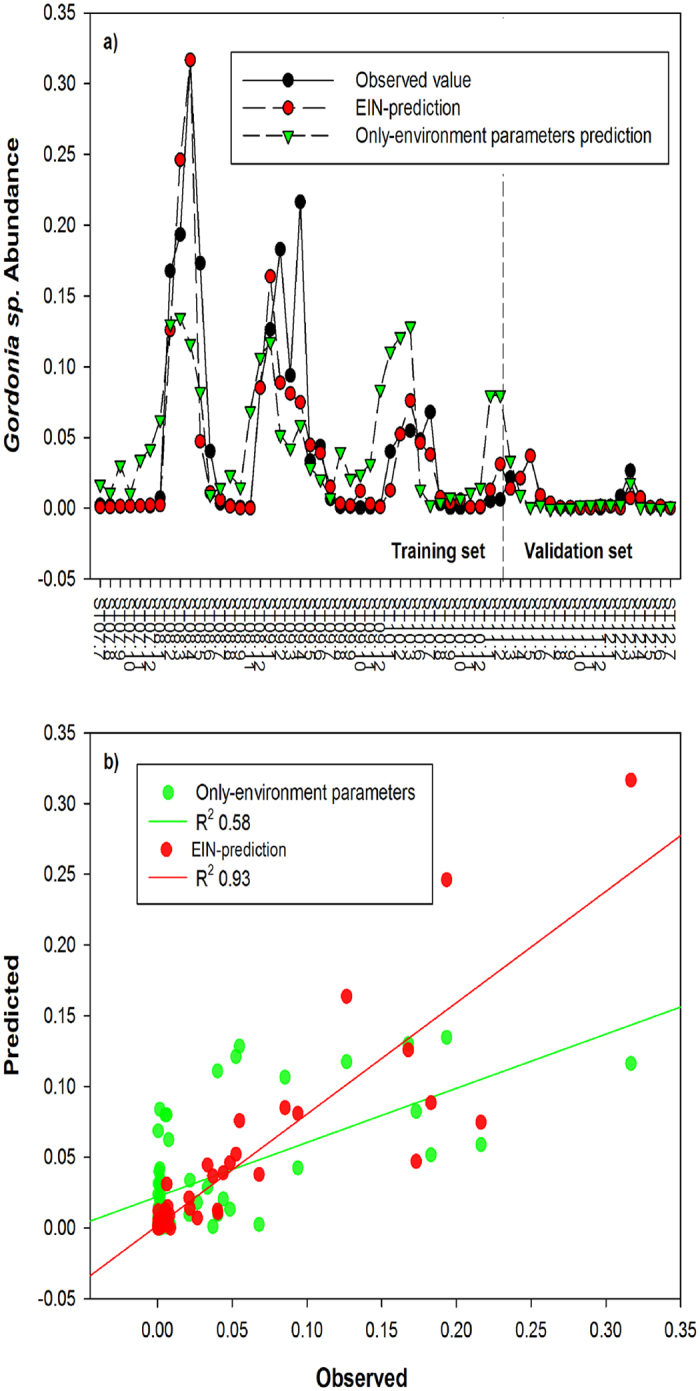
Model *Gordonia* sp. dynamics with EIN: (**a**) Observed and predicted abundance of *Gordonia* sp. along the five years, model constructed with the first 45 samples and validated with the left 13 samples; (**b**) Observed *Gordonia* sp. abundance against model predicted abundance, regression R^2^ value for EIN module and only environmental parameters module were given.

Since correlation based analysis can only capture potential linear or rank linear relationships between BFB and the related abiotic and biotic factors, to explore possible non-linear relationships of them, we constructed EIN which was a Bayesian network generated with environmental parameters, interactions between BFB and other bacteria. The Bayesian network was probabilistic graphical model which represented conditional dependence relationships among a group of random variables. Thus, edges in the network possibly referred to causal-relationships between the parent node and the children node. BFB with frequency less than 75% of total samples were filtered, which selected three BFB and other related bacteria as well as all the environmental/operational parameters for the model input. To extrapolate the model along time, the 58 samples dataset was divided into training set (45 samples, 75% of the 58 samples) and a validation set (13 samples, 25%). EIN were constructed with the training set, and then the derived functions for BFB from Erequa^0.9^ were validated with the validation set. To compare the models with and without biotic interactions, another model was also constructed with only the operation/environmental parameters and BFB. Results of the derived functions for the three selected BFB and their accuracies were listed in Table S2. Figure 5a showed the predicted and observed *Gordonia* sp. along time and the predicted values were plotted against observed values in Fig. 5b. The figure showed that the prediction accuracy of EIN derived function was much better compared with the function derived only by the environmental parameters. Prediction accuracy was fairly good with an R^2^ of 0.93 between observed and calculated abundance and a mean square error (MSE) only 7.41e-06. *Mycobacterium* sp. also followed the same trend and fitted the model well (Fig. S3, Table S2). For both EIN model and environmental/operational parameters only model, Nostocodia limicola II *Tetraspheara* sp. only correlated with temperature and the prediction accuracy was high with a R^2^ value of 0.93, indicating the high dependence of *Tetraspheara* sp. on temperature.

Our current knowledge maybe not enough to explain the relationships among the parent bacteria species and environmental parameters with BFB in the EIN model, however, the derived function from the anterior time series (training set from 07-2007 ~ 02-2011) could be fairly good fitted in the posterior time (validation set from 03-2011 ~ 07-2-12), indicating the extrapolation ability of the model. It should be noticed that the related bacteria with BFB in the EIN module were not exactly identical to those identified using the Spearman correlation based network. This was mainly due to the ability of the Bayesian inference in identifying nonlinear relationship among variables. Overall, the incorporation of biotic interaction in the EIN model had better accuracy than that one with only environmental parameters indicating that the biotic factors were also important factors in determination of the population of BFB.

## Conclusions

In conclusion, pyrosequencing of 16S rRNA gene revealed high diversity (17 types) of bulking and foaming bacteria occurring in a full scale WWTP in five years, occupying 0.6~36% (averagely 8.52% ± 7.3%) total bacteria in the activated sludge system. Total BFB showed significant seasonal variations with higher abundance in winter than summer and the variation was mainly contributed by *Gordonia* sp. The *Gordonia* sp. was positively correlated with NO_2_-N and negatively correlated with NO_3_-N, and *Tetrasphaera* sp. was negatively correlated with temperature and positively correlated with NH_3_-N in activated sludge. CCA showed consistent results with the above correlation analysis. Bacteria correlated with BFB could be clustered into two modules; the two modules were negatively correlated with each other and positively correlated inside each module. Correlations between *Gordonia* sp. and other bacteria indicated that over 90% of the strong correlations (mainly from phyla *Proteobacteria* and *Chloroflexi*) were negative and the phylum TM_7 were all positively correlated with BFB. Finally, we demonstrated that EIN could be applied in the artificial engineering system of AS to predict BFB.

## Methods

### Sampling and data sets

The AS samples were monthly collected from the aeration tank of a full-scale WWTP performing anoxic/oxic process with a proceeding ability of 216,000 m^3^ day^−1^, as described before^22^ over five years from 2007 to 2012. The AS was 1:1 mixed with absolute ethanol and stored in −20 °C fridge before DNA extraction^22^. Wastewater characteristics and operational parameters were collected accordingly from Drainage Services Department.

### Sequence process and identification of bulking and foaming bacteria

DNA extraction, PCR amplification and 454 pyrosequencing were conducted as described before^22^. Raw sequences from Roche 454 FLX Titanium platform were processed with QIIME pipeline 1.70*v*^32^. Firstly, the raw sequences were de-noised by QIIME denoiser^33^. Then ChimeraSlayer algorithm^34^ was used to identify chimera sequences. After removing chimera sequences, the clean sequences of each sample were normalized to 6,000. To identify BFB in each sample, BLAST 2.28+ was used to search BFB in each sample against the BFB database^15^ with a similarity cut off of 97% and minimum alignment length of 300 bps. To investigate bacterial species correlated with BFB, we firstly remove all the BFB sequences and then the remaining sequences were clustered into operational taxonomy units (OTUs) at 0.97 cut-off with UCLUST^35^. Representative sequence of each OTU was then sent to RDP Classifier 2.1^36^ for taxonomy identification. QIIME was used to generate the OTUs table for all samples. OTUs were filtered by abundance and frequencies, any OTU with less than 5 pyrotags and frequency lower than 50% (present in <50% of samples) were removed. Then remaining OTUs were used to calculate Spearman correlation with BFB. Those with Spearman coefficient value > 0.6 or <−0.6 (*P*-value < 0.01) were retained as bacterial species which were strongly correlated with BFB^19^.

### Statistical analysis and network analysis

Time series heat-map of BFB was generated with function ‘heatmap.2’ in R3.0^37^ package ‘gplots’. One way ANOVA and post-hoc Tukey HSD tests on summer-autumn and winter-spring were conducted with R package. Spearman and Pearson correlation analysis were conducted to identify those operational parameters and water/sludge quality parameters which showed strong relationships with BFB. Pearson and Spearman coefficient index were calculated with function ‘rcorr’ in R3.0 package ‘Hmisc’.The Cytoscape3.0^38^ was applied to generate the network between BFB and their correlated bacterial OTUs. The Spring-Embedded layout algorithm on edge value was used to cluster OTUs and BFB in the network. Canonical corresponding analysis (CCA) was generated by Canoco4.5.

### EIN construction and functions generation with Erequa

An EIN was a Bayesian network (BN) with both environmental parameters and microbial interactions as proposed in a study using EIN to predict the microbial community of ocean with time series data^21^. To construct the EIN, all environmental parameters, selected OTUs and BFB were merged into one matrix; then this matrix was sent to learn the BN by Bayesian Network Inference with Java Objects (BANJO) v2.1^39^ (http://www.cs.duke.edu/~amink/software/banjo/). Due to different units for environmental parameters, all the environmental parameters were transformed to 1 to 100 by the following equation for normalization^21^


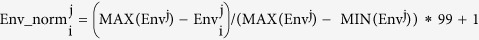


where 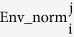 is the normalized value for parameter j at time i, 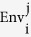 is the observed value, MAX and MIN give the maximum and minimum values for parameter j across all time points.

OTUs and BFB were all using relative abundance in the matrix. OTUs and BFB were selected by a standard that the average abundance should be larger than 0.01% and the presence across samples should be larger than 75%. After filtering, only the most abundant three BFB met the requirement. For the detail running parameters of BANJO, a maximum of five parents, All Local Moves proposer, simulated annealing and randomly configured networks were used. Since we only concerned about BFB dynamics and their determinative factors, so only edges from environmental parameters and other OTUs to BFB were allowed. A consensus network was generated from all the learned networks by BANJO. The EIN we finally obtained was a directed acyclical graph (DAG) whose edges represented causal relationship between the parent nodes and their child nodes inferred by the observed data. The relationship in the EIN could be seen as an artificial neural network. Then each BFB can be expressed as a function of its parent nodes. The function was deduced using Eureqa^0.9^ beta software^40^. Eureqa^0.9^ could deduce equations of variables in numerical dataset without prior knowledge about the system. As Eureqa^0.9^ generated several functions fitting the data, the final function chosen was a trade-off between the complexity of the function and its fitness which was measured by Pearson’s coefficient.

## Additional Information

**How to cite this article**: Jiang, X.-T. *et al.* Population Dynamics of Bulking and Foaming Bacteria in a full-scale Wastewater Treatment Plant over Five Years. *Sci. Rep.* **6**, 24180; doi: 10.1038/srep24180 (2016).

## Supplementary Material

Supplementary Information
