# Supplementary material for: Population Dynamics of Bulking and Foaming Bacteria in a Full-scale Wastewater Treatment Plant over Five Years
Source: Sci Rep. 2016 Apr 11;6:24180. doi: 10.1038/srep24180 (PMC4827064; doi:10.1038/srep24180)
Supplement: Supplementary Information [file srep24180-s1.pdf]

Supplementary Information:

Population Dynamics of Bulking and Foaming Bacteria in a full-scale Wastewater Treatment Plant over Five Years

Xiao-Tao Jiang, Feng Guo and Tong Zhang*

Author affiliation: Environmental Biotechnology Lab, The University of Hong Kong SAR China;

Corresponding author: Dr. Tong Zhang (Associate Professor)
E-mail: zhangt@hku.hk
Address: Environmental Biotechnology Lab, the University of Hong Kong, Pokfulam Road, Hong Kong
Tel: 852-28591968 (lab), 28578551 (office) Fax: 852-25595337

This supplementary information contains:

Supplementary figure S1
Supplementary figure S2
Supplementary figure S3
Supplementary figure S4
Supplementary figure S5

Supplementary table S1
Supplementary table S2

Supplementary figure 1: Histogram of the percentage distribution of the most abundant six BFB in Shatin wastewater treatment plant over five years. The X-axis was the percentage of BFB over total bacteria; and the Y-axis depicts the frequencies distribution. The 'x' value in each figure was the average percentage of that BFB in total bacteria.

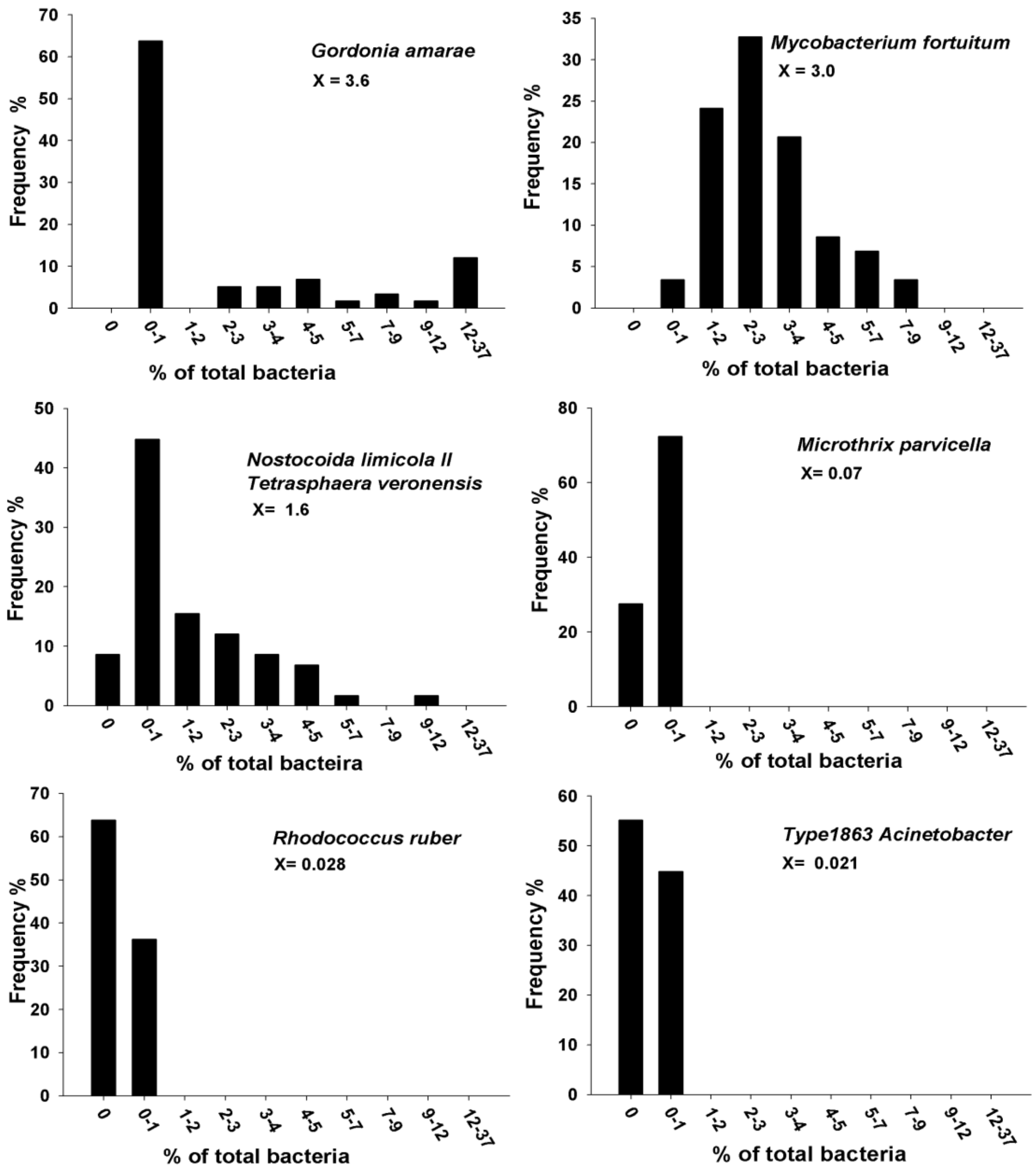

Supplementary figure 2: Canonical corresponding analysis of the dominant BFB and operational parameters in Shatin WWTP. Triangles were BFB, dots were samples, the blue arrows were influent parameters and the red arrows were operational and activated sludge quality parameters. Red dots represented the summer-autumn samples and black dots were the winter-spring samples.

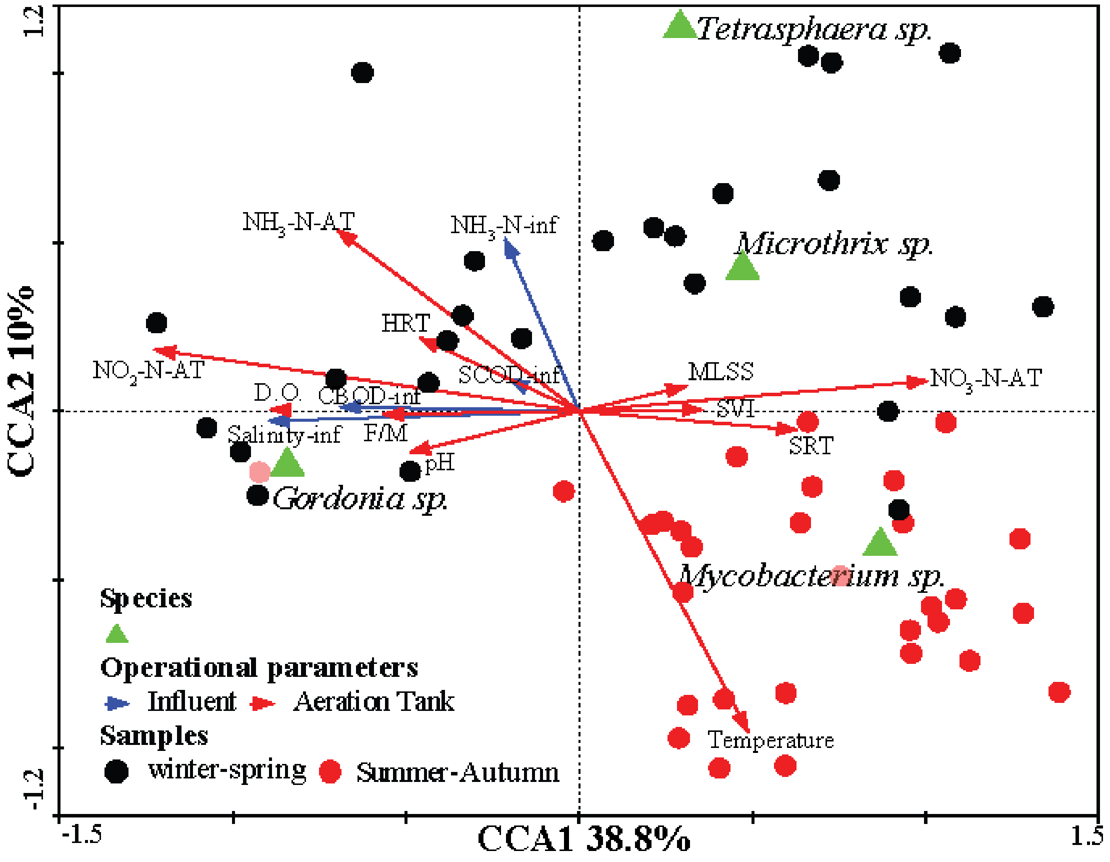

Supplementary figure 3: a1, b1) comparison of observed abundance and the predicted abundance of *Mycobacteriu sp.* and *Tetraspheara sp.*; a2, b2) Regression of the observed against the predicted abundance for *Mycobacteriu sp.* and *Tetraspheara sp.* for EIN based model and the only environmental parameters based model.

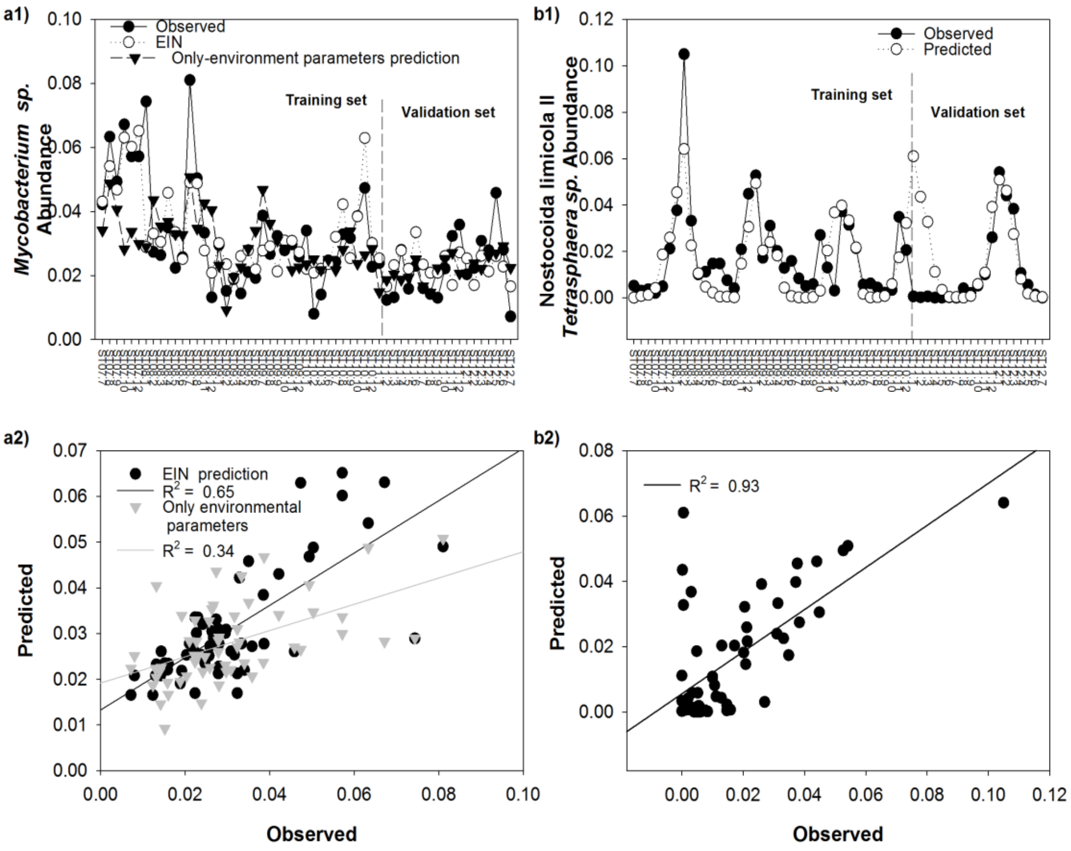

Supplementary figure 4: Nitrogen metabolism KEGG pathway annotation of reconstructed *Gordonia sp.*bin from Shatin foaming sample. Red rectangle represent genes detected in the bin.

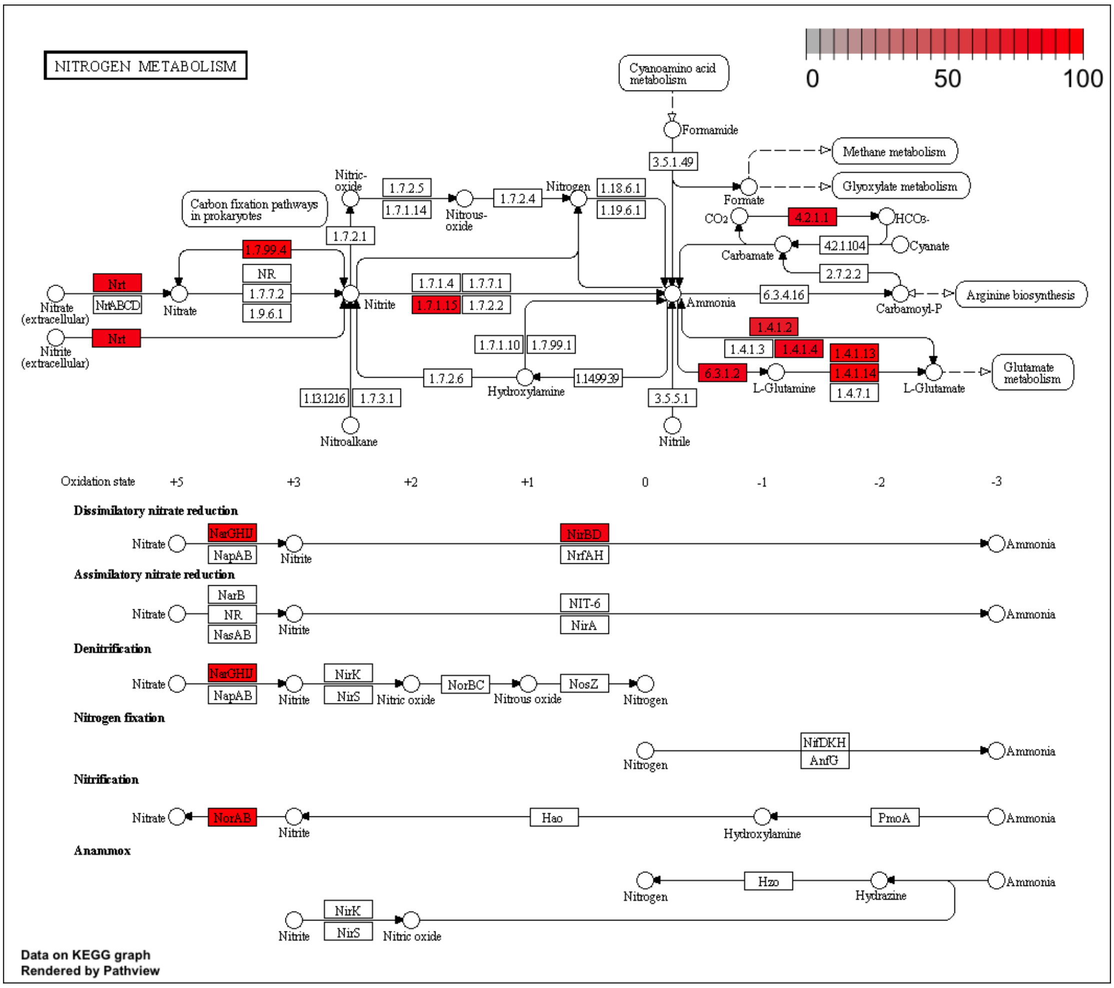

Supplementary figure 5: BFB dynamics along with temperature over five years, the Hong Kong was a tropic climate aera with temperature variant from 12 ºC to 32 ºC over the sampling period.

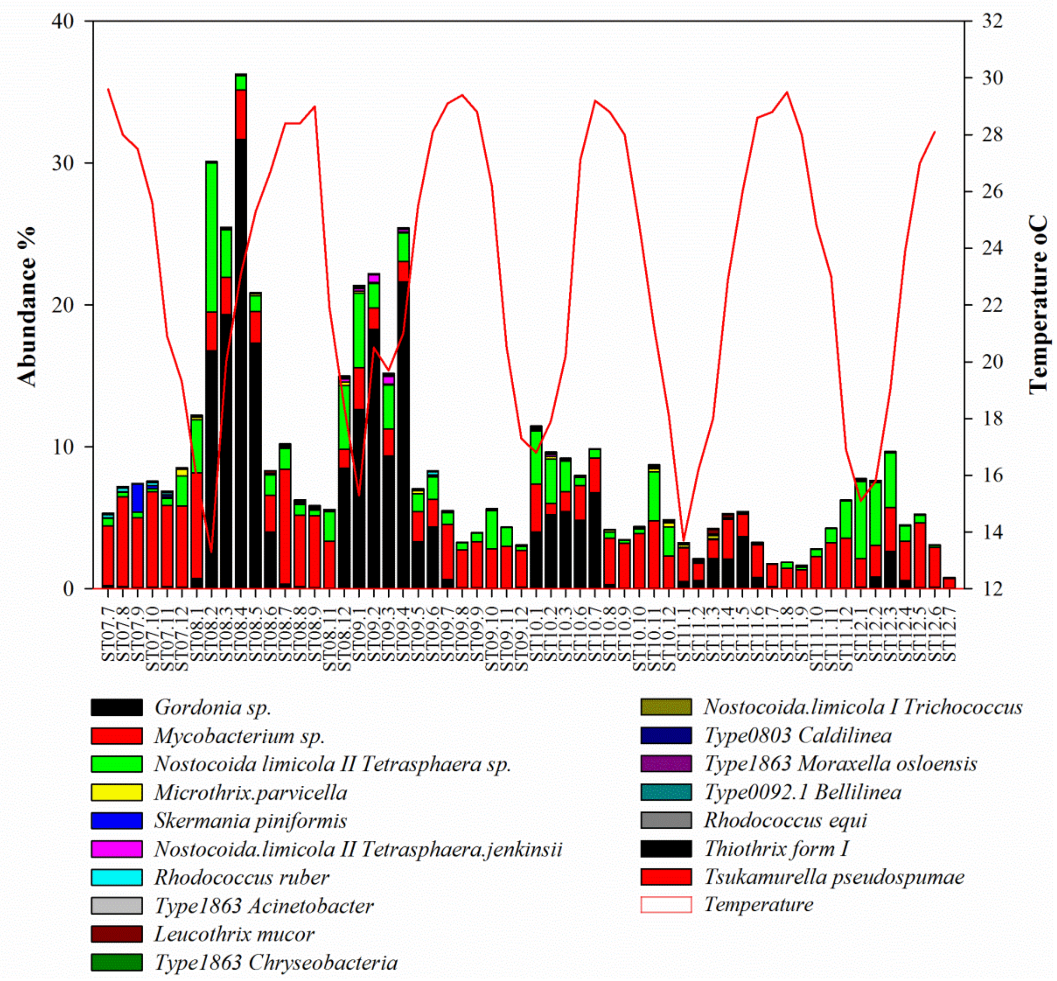

Supplementary table S1 Bacteria species directly correlated with bulking and foaming species. OTUs were generated with QIIME pipeline by a cut off 0.97. The taxonomy assignments of OTUs were conducted with RDP classifier 2.1. Spearman correlations were calculated (only significantly correlated OTUs were listed with correlation coefficient over than 0.6 or -0.6, P-value < 0.01) and the relative abundance of each OTU was listed. Modules was colored according to the module in network, green cells were belong to module 1 which were most negatively correlated with BFB and red cells were module 2, which were most positively correlated with BFB.

Bulking and foaming species	OTU_id	Spearman correlation coefficient	Module	Relative abundance of species	Lowest taxonomy annotation of OTUs
	denovo5212	-0.7124	Module1	0.0050	k__Bacteria;p__Spirochaetes;c__[Leptospirae];o__[Leptospirales];f__Leptospiraceae
	denovo4985	-0.6450	Module1	0.0015	k__Bacteria;p__Proteobacteria;c__Deltaproteobacteria;o__Myxococcales;f__Nannocystaceae;g__Plesiocystis
	denovo2769	-0.6040	Module1	0.0003	k__Bacteria;p__Proteobacteria;c__Deltaproteobacteria;o__Myxococcales
	denovo4323	-0.6331	Module1	0.0004	k__Bacteria;p__Proteobacteria;c__Deltaproteobacteria
	denovo3000	-0.6416	Module1	0.0014	k__Bacteria;p__Proteobacteria;c__Deltaproteobacteria
	denovo902	-0.6069	Module1	0.0040	k__Bacteria;p__Proteobacteria;c__Betaproteobacteria;o__Burkholderiales;f__Comamonadaceae;g__Rubrivivax
	denovo3846	0.6543	Module2	0.0059	k__Bacteria;p__Proteobacteria;c__Betaproteobacteria;o__Burkholderiales;f__Comamonadaceae
	denovo3064	-0.6227	Module1	0.0010	k__Bacteria;p__Proteobacteria;c__Alphaproteobacteria;o__Rickettsiales
	denovo4657	-0.6001	Module1	0.0098	k__Bacteria;p__Proteobacteria;c__Alphaproteobacteria;o__Rhizobiales;f__Hyphomicrobiaceae
	denovo5373	-0.6127	Module1	**0.0029**	k__Bacteria;p__Proteobacteria;c__Alphaproteobacteria;o__Rhizobiales;f__Hyphomicrobiaceae
	denovo1264	-0.6162	Module1	0.0012	k__Bacteria;p__Proteobacteria;c__Alphaproteobacteria;o__Rhizobiales;f__Hyphomicrobiaceae
	denovo3900	-0.6391	Module1	0.0034	k__Bacteria;p__Proteobacteria;c__Alphaproteobacteria;o__Rhizobiales
	denovo3702	-0.6524	Module1	0.0048	k__Bacteria;p__Proteobacteria;c__Alphaproteobacteria;o__Rhizobiales
	denovo4694	-0.7147	Module1	0.0019	k__Bacteria;p__Proteobacteria;c__Alphaproteobacteria;o__Rhizobiales
	denovo4665	-0.7588	Module1	0.0012	k__Bacteria;p__Proteobacteria;c__Alphaproteobacteria;o__Rhizobiales
	denovo485	-0.7576	Module1	0.0010	k__Bacteria;p__GOUTA4

Gordonia sp.	denovo5366	-0.6695	Module1	0.0008	k__Bacteria;p__Gemmatimonadetes;c__Gemm-2
	denovo3317	-0.7032	Module1	0.0037	k__Bacteria;p__Chloroflexi;c__TK17;o__mle1-48
	denovo1256	0.6353	Module2	0.0048	k__Bacteria;p__Chloroflexi;c__Thermomicrobia;o__JG30-KF-CM45
	denovo2176	-0.6662	Module1	0.0046	k__Bacteria;p__Chloroflexi;c__Thermomicrobia;o__JG30-KF-CM45
	denovo5253	-0.6744	Module1	0.0011	k__Bacteria;p__Chloroflexi;c__Thermomicrobia;o__JG30-KF-CM45
	denovo3937	-0.7708	Module1	0.0043	k__Bacteria;p__Chloroflexi;c__Thermomicrobia;o__JG30-KF-CM45
	denovo4864	-0.6041	Module1	0.0002	k__Bacteria;p__Chloroflexi;c__Anaerolineae;o__DRC31
	denovo1660	-0.6926	Module1	0.0115	k__Bacteria;p__Chloroflexi;c__Anaerolineae;o__Caldilineales;f__Caldilineaceae
	denovo933	-0.7275	Module1	0.0009	k__Bacteria;p__Chloroflexi;c__Anaerolineae
	denovo907	-0.7219	Module1	0.0015	k__Bacteria;p__Chlorobi;c__OPB56
	denovo4231	-0.6060	Module1	0.0019	k__Bacteria;p__Bacteroidetes;c__Sphingobacteriia;o__Sphingobacteriales;f__Saprospiraceae
	denovo6163	-0.6607	Module1	0.0031	k__Bacteria;p__Bacteroidetes;c__Sphingobacteriia;o__Sphingobacteriales;f__Saprospiraceae
	denovo1999	-0.6971	Module1	0.0489	k__Bacteria;p__Bacteroidetes;c__Sphingobacteriia;o__Sphingobacteriales;f__Saprospiraceae
	denovo1813	-0.6063	Module1	0.0080	k__Bacteria;p__Bacteroidetes;c__Sphingobacteriia;o__Sphingobacteriales;f__Ekhidnaceae
	denovo5559	0.7033	Module2	0.0161	k__Bacteria;p__Bacteroidetes;c__Flavobacteriia;o__Flavobacteriales;f__Flavobacteriaceae
	denovo2902	-0.6430	Module1	0.0007	k__Bacteria;p__Bacteroidetes;c__Flavobacteriia;o__Flavobacteriales;f__Flavobacteriaceae
	denovo1471	-0.6104	Module1	0.0272	k__Bacteria;p__Actinobacteria;c__Thermoleophilia;o__Solirubrobacterales
	denovo6096	-0.8059	Module1	0.0095	k__Bacteria;p__Actinobacteria;c__Thermoleophilia;o__Solirubrobacterales
	denovo3495	-0.6243	Module1	0.0064	k__Bacteria;p__Actinobacteria;c__Actinobacteria;o__Actinomycetales;f__Nocardioidaceae
	denovo5219	-0.6010	Module1	0.0105	k__Bacteria;p__Actinobacteria;c__Actinobacteria;o__Actinomycetales;f__Mycobacteriaceae;g__Mycobacterium
	denovo4548	0.9800	Module2	0.0888	k__Bacteria;p__Actinobacteria;c__Actinobacteria;o__Actinomycetales;f__Gordoniaceae;g__Gordonia
	denovo1055	-0.6985	Module1	0.0022	k__Bacteria;p__Actinobacteria;c__Acidimicrobiia;o__Acidimicrobiales
	denovo5028	-0.6908	Module1	0.0005	k__Bacteria;p__Acidobacteria;c__Solibacteres;o__Solibacterales;f__Solibacteraceae

	denovo1588	-0.6078	Module1	0.0014	k__Bacteria
	denovo2335	-0.7839	Module1	0.0039	k__Bacteria
Microthrix sp.	denovo342	-0.6487	Module1	0.0004	k__Bacteria;p__Proteobacteria;c__Deltaproteobacteria
	denovo902	-0.6443	Module1	0.0040	k__Bacteria;p__Proteobacteria;c__Betaproteobacteria;o__Burkholderiales;f__Comamonadaceae;g__Rubrivivax
	denovo4944	-0.6093	Module1	0.0011	k__Bacteria;p__Cyanobacteria;c__4C0d-2;o__SM1D11
	denovo4548	0.6053	Module2	0.0888	k__Bacteria;p__Actinobacteria;c__Actinobacteria;o__Actinomycetales;f__Gordoniaceae;g__Gordonia
	denovo4572	0.9248	Module2	0.0019	k__Bacteria;p__Actinobacteria;c__Acidimicrobiia;o__Acidimicrobiales;f__Microthrixaceae
	denovo5028	-0.6126	Module1	0.0005	k__Bacteria;p__Acidobacteria;c__Solibacteres;o__Solibacterales;f__Solibacteraceae
Mycobacterium sp.	denovo3870	0.6583	Module2	0.0458	k__Bacteria;p__Chloroflexi;c__Thermomicrobia;o__JG30-KF-CM45
	denovo726	0.6434	Module1	0.0006	k__Bacteria;p__Chloroflexi;c__Anaerolineae;o__SBR1031;f__A4b
	denovo4565	0.6170	Module2	0.0143	k__Bacteria;p__Actinobacteria;c__Actinobacteria;o__Actinomycetales;f__Nocardioidaceae
	denovo3977	0.8681	Module1	0.0863	k__Bacteria;p__Actinobacteria;c__Actinobacteria;o__Actinomycetales;f__Mycobacteriaceae;g__Mycobacterium
	denovo2387	0.7976	Module2	0.0006	k__Bacteria;p__Proteobacteria;c__Alphaproteobacteria;o__Rhodospirillales
	denovo3218	0.7753	Module2	0.0002	k__Bacteria;p__Proteobacteria;c__Deltaproteobacteria
	denovo606	0.7403	Module2	0.0004	k__Bacteria;p__Proteobacteria;c__Alphaproteobacteria;o__Rhodospirillales
	denovo1101	0.6835	Module2	0.0002	k__Bacteria;p__Proteobacteria;c__Alphaproteobacteria;o__Rhizobiales
	denovo5666	0.6751	Module2	0.0011	k__Bacteria;p__Proteobacteria;c__Alphaproteobacteria;o__Rhodobacterales;f__Rhodobacteraceae;g__Paracoccus
	denovo3353	0.6641	Module2	0.0002	k__Bacteria;p__Proteobacteria;c__Alphaproteobacteria;o__Sphingomonadales
	denovo5610	0.6594	Module2	0.0005	k__Bacteria;p__Chloroflexi;c__Anaerolineae;o__Caldilineales;f__Caldilineaceae
	denovo1722	0.6561	Module2	0.0007	k__Bacteria;p__Bacteroidetes;c__Flavobacteriia
	denovo3097	0.6516	Module2	0.0003	k__Bacteria;p__Bacteroidetes;c__Sphingobacteriia;o__Sphingobacteriales;f__Saprospiraceae
	denovo5472	0.6448	Module2	0.0007	k__Bacteria;p__TM7;c__TM7-1

**Nostocoida.limicolaII *Tetrasphaera.jenkinsii***	denovo6046	0.6364	Module2	0.0004	k__Bacteria;p__Proteobacteria;c__Alphaproteobacteria;o__BD7-3
	denovo5587	0.6353	Module2	0.0005	k__Bacteria;p__Actinobacteria;c__Actinobacteria;o__Actinomycetales;f__Nocardioidaceae;g__Nocardioides
	denovo3442	0.6303	Module2	0.0082	k__Bacteria;p__Chloroflexi;c__Anaerolineae;o__c26
	denovo3989	0.6151	Module2	0.0002	k__Bacteria;p__Proteobacteria;c__Alphaproteobacteria;o__BD7-3
	denovo2754	0.6018	Module2	0.0004	k__Bacteria;p__Proteobacteria;c__Deltaproteobacteria
	denovo3055	-0.6007	Module1	0.0062	k__Bacteria;p__Proteobacteria;c__Alphaproteobacteria;o__Rhizobiales
	denovo2284	-0.6045	Module1	0.0006	k__Bacteria;p__Proteobacteria;c__Alphaproteobacteria;o__Rhizobiales;f__Hyphomicrobiaceae;g__Rhodoplanes
	denovo3641	-0.6115	Module1	0.0036	k__Bacteria;p__Proteobacteria;c__Alphaproteobacteria;o__Rhizobiales;f__Hyphomicrobiaceae
	denovo2529	-0.6177	Module1	0.0040	k__Bacteria;p__Chloroflexi;c__TK17;o__mle1-48
	denovo3833	-0.6450	Module1	0.0291	k__Bacteria;p__Proteobacteria;c__Alphaproteobacteria;o__Rhizobiales;f__Rhodobiaceae
	denovo2161	-0.6480	Module1	0.0051	k__Bacteria;p__Actinobacteria;c__Actinobacteria;o__Actinomycetales;f__Mycobacteriaceae;g__Mycobacterium
	denovo1024	-0.6671	Module1	0.0126	k__Bacteria;p__Proteobacteria;c__Gammaproteobacteria;o__Xanthomonadales;f__Xanthomonadaceae;g__Dokdonell[illegible]
	denovo1480	-0.6823	Module1	0.0008	k__Bacteria;p__Proteobacteria;c__Alphaproteobacteria;o__Rhizobiales
**Nostocoida.limicolaII *Tetrasphaera sp.***	denovo2524	0.8018	Module2	0.0512	k__Bacteria;p__Actinobacteria;c__Actinobacteria;o__Actinomycetales;f__Intrasporangiaceae
	denovo1861	0.7005	Module2	0.0080	k__Bacteria;p__Proteobacteria;c__Alphaproteobacteria;o__Rhizobiales;f__Rhizobiaceae
	denovo1139	0.6970	Module2	0.0045	k__Bacteria;p__Actinobacteria;c__Actinobacteria;o__Actinomycetales;f__Microbacteriaceae;g__Leucobacter
	denovo2927	0.6716	Module2	0.0043	k__Bacteria;p__Bacteroidetes;c__Sphingobacteriia;o__Sphingobacteriales;f__Saprospiraceae
	denovo5234	0.6375	Module2	0.0033	k__Bacteria;p__TM7
	denovo2695	0.6339	Module2	0.0054	k__Bacteria;p__TM7;c__TM7-3
	denovo124	0.6112	Module2	0.0023	k__Bacteria;p__Proteobacteria;c__Alphaproteobacteria;o__Rhodobacterales;f__Rhodobacteraceae;g__Paracoccus
	denovo1436	0.6053	Module2	0.0016	k__Bacteria;p__Bacteroidetes;c__Sphingobacteriia;o__Sphingobacteriales;f__Saprospiraceae
	denovo1124	-0.6548	Module1	0.0008	k__Bacteria;p__Proteobacteria;c__Alphaproteobacteria;o__BD7-3

	denovo4534	0.9436	Module2	0.0010	k__Bacteria;p__Actinobacteria;c__Actinobacteria;o__Actinomycetales;f__Nocardiaceae;g__Rhodococcus;s__ruber
	denovo4565	0.7522	Module2	0.0143	k__Bacteria;p__Actinobacteria;c__Actinobacteria;o__Actinomycetales;f__Nocardioidaceae
	denovo5114	0.7442	Module2	0.0003	k__Bacteria;p__Proteobacteria;c__Alphaproteobacteria;o__Rhodobacterales;f__Rhodobacteraceae
	denovo2432	0.7359	Module2	0.0015	k__Bacteria;p__TM7
	denovo4039	0.6989	Module2	0.0002	k__Bacteria;p__Firmicutes;c__Clostridia;o__Clostridiales;f__Ruminococcaceae
	denovo3091	0.6965	Module2	0.0005	k__Bacteria;p__Actinobacteria;c__Actinobacteria;o__Actinomycetales
	denovo1862	0.6928	Module2	0.0006	k__Bacteria;p__Actinobacteria;c__Actinobacteria;o__Actinomycetales;f__Beutenbergiaceae
	denovo2199	0.6795	Module2	0.0003	k__Bacteria;p__GN02;c__3BR-5F
Rhodococcus.ruber	denovo4632	0.6734	Module2	0.0410	k__Bacteria;p__Chloroflexi;c__Anaerolineae
	denovo5564	0.6701	Module2	0.0785	k__Bacteria;p__Proteobacteria;c__Alphaproteobacteria;o__Rhodobacterales;f__Rhodobacteraceae
	denovo5083	0.6647	Module2	0.0004	k__Bacteria;p__Proteobacteria;c__Alphaproteobacteria;o__Rhodobacterales;f__Rhodobacteraceae
	denovo1172	0.6481	Module2	0.0011	k__Bacteria
	denovo5223	0.6421	Module2	0.0058	k__Bacteria;p__Chloroflexi;c__Anaerolineae;o__Caldilineales;f__Caldilineaceae
	denovo4358	0.6387	Module2	0.0112	k__Bacteria;p__Chloroflexi;c__Anaerolineae;o__SBR1031;f__A4b
	denovo1790	0.6181	Module2	0.0041	k__Bacteria;p__Chloroflexi;c__Anaerolineae;o__SBR1031;f__A4b
	denovo55	0.6153	Module2	0.0002	k__Bacteria;p__Proteobacteria;c__Deltaproteobacteria
	denovo3015	0.6125	Module2	0.0006	k__Bacteria;p__Chloroflexi;c__Anaerolineae;o__Caldilineales;f__Caldilineaceae
	denovo1810	0.6080	Module2	0.0003	k__Bacteria;p__Actinobacteria;c__Actinobacteria;o__Actinomycetales;f__Mycobacteriaceae;g__Mycobacterium
	denovo4951	0.6030	Module2	0.0130	k__Bacteria;p__TM7;c__TM7-1
	denovo518	-0.6203	Module1	0.0013	k__Bacteria;p__Proteobacteria;c__Gammaproteobacteria;o__Alteromonadales;f__OM60
Type1863Acinetobacter	denovo1953	-0.6006	Module2	0.0032	k__Bacteria;p__Actinobacteria;c__Actinobacteria;o__Actinomycetales
Type1863Chryseobacteria	denovo4157	0.6206	Module1	0.0004	k__Bacteria;p__Bacteroidetes;c__Flavobacteriia;o__Flavobacteriales;f__Flavobacteriaceae

Supplementary table S2: The derived function by Eureqa 1.03 beta for the most abundant three bulking and foaming species. Abbreviations: EIN (environmental interaction network), MSE (Mean squared error), NO_2-N (Nitrite concentration in aeration tank), NO_3-N (Nitrate concentration in aeration tank), NH_3-N (Ammonia concentration in aeration tank), MLSS (Mixed liquor suspended solids), Salinity-inf (Salinity of influent). The lowest classification of these associated OTUs were denovo524 (*f__Phyllobacteriaceae*), denovo2281 (*f__Flavobacteriaceae*), denovo2850 (*o__JG30-KF-CM45*), denovo2277 (*o__Clostridiales*).

Predicted with EIN			Predicted with only environmental parameters		
**Function**	**MSE**	**R^2**	**Function**	**MSE**	**R^2**
Gordonia sp. = 0.02 + 4.51*denovo2281 + 1751.84*denovo524*denovo2850 - 2.37e-6*(NH_3-N)*(NO_2-N) - 106.09*denovo2281^2	7.41E-06	0.93	***Gordonia sp.*** = 0.0028 + 0.0013*(NO_3-N) + 2.98e-10*(NO_2-N)*(NO_3-N)^3 + 2.91e-10*(NO_3-N)*(NO_2-N)^3 - 1.68e-7*(NH_3-N)*(NO_2-N)*(NO_3-N)	9.74E-05	0.58
Mycobacterium sp. = 29.69*denovo2277^2 + 0.03*cos(sin(14.60/denovo2277))	5.26E-05	0.65	***Mycobacterium sp.*** = 0.013 + 7.61e-5*MLSS + 3.84e-6*(Salinity-inf)*MLSS + 1.05e-6*(Salinity-inf)^2 - 6.52e-8*(Salinity-inf)	6.44E-05	0.31
**Nostocoida limicola II *Tetrasphaera sp.*** = 6.4e-6-6*Termperature^2				2.38E-05	0.93
